# First description and validation of a new method for estimating aortic stenosis burden and predicting the functional response to TAVI

**DOI:** 10.3389/fcvm.2023.1215826

**Published:** 2023-11-14

**Authors:** Jose M. de la Torre Hernandez, Gabriela Veiga Fernandez, Eyal Ben-Assa, Julia Iribarren, Fermin Sainz Laso, Dae-Hyun Lee, Cristina Ruisanchez Villar, Piedad Lerena, Tamara Garcia Camarero, Jose L. Iribarren Sarrias, Jose M. Cuesta Cosgaya, Maria E. Maza Fernandez, Celia Garilleti, Victor Fradejas-Sastre, Mercedes Benito, Sergio Barrera, Aritz Gil Ongay, Jose A. Vazquez de Prada, Javier Zueco

**Affiliations:** ^1^ Cardiology Division, Hospital Universitario Marques de Valdecilla, IDIVAL, Santander, Spain; ^2^ Department of Cardiology, Medical School, University of Cantabria, Santander, Spain; ^3^ Cardiology Division, Assuta Ashdod University Hospital, Ben Gurion University, Ashdod, Israel; ^4^ School of Mathematics, Universidad de la Laguna, San Cristobal de la Laguna, Spain; ^5^ Intensive Care Unit, Complejo Hospitalario Universitario de Canarias, Santa Cruz de Tenerife, Spain; ^6^ Hydrodynamics and Coastal Infrastructures Group of IH Cantabria, Instituto de Hidraulica Ambiental, Universidad de Cantabria, Santander, Spain

**Keywords:** aortic stenosis, transcatheter aortic valve implantation, clinical outcomes, aortic pressure, flow velocity, left ventricular outflow tract

## Abstract

**Background:**

Up to one-fifth of patients continue to have poor quality of life after transcatheter aortic valve implantation (TAVI), with an additional similar proportion not surviving 1 year after the procedure. We aimed to assess the value of a new method based on an integrated analysis of left ventricular outflow tract flow velocity and aortic pressure to predict objective functional improvement and prognosis after TAVI.

**Methods:**

In a cohort of consecutive patients undergoing TAVI, flow velocity–pressure integrated analysis was obtained from simultaneous pressure recordings in the ascending aorta and flow velocity recordings in the left ventricular outflow tract by echocardiography. Objective functional improvement 6 months after TAVI was assessed through changes in a 6-min walk test and NT-proBNP levels. A clinical follow-up was conducted at 2 years.

**Results:**

Of the 102 patients studied, 82 (80.4%) showed objective functional improvement. The 2-year mortality of these patients was significantly lower (9% vs. 44%, *p* = 0.001). In multivariate analysis, parameter “(Pressure at Vmax − Pressure at Vo)/Vmax” was found to be an independent predictor for objective improvement. The C-statistic was 0.70 in the overall population and 0.78 in the low-gradient subgroup. All echocardiographic parameters and the valvuloarterial impedance showed a C-statistic of <0.6 for the overall and low-gradient patients. In a validation cohort of 119 patients, the C-statistic was 0.67 for the total cohort and 0.76 for the low-gradient subgroup.

**Conclusion:**

This new method allows predicting objective functional improvement after TAVI more precisely than the conventional parameters used to assess the severity of aortic stenosis, particularly in low-gradient patients.

## Introduction

Transcatheter aortic valve implantation (TAVI) substantially improves survival and quality of life in most patients with severe aortic stenosis (AS). Nonetheless, up to one-fifth of patients continue to have poor quality of life after TAVI, with an additional similar proportion not surviving 1 year after the procedure (1). However, given the poor prognosis associated with non-procedural management of symptomatic severe AS, the decision will usually be made to proceed with TAVI, even if there is a concern for a sub-optimal result (2).

Nonetheless, it is important to explore and understand the factors associated with a sub-optimal outcome to inform decisions regarding the optimal timing of TAVI and/or adjunctive interventions that may improve the outcomes after TAVI such as particular medications and rehabilitation. Furthermore, the identification of such predictors is important to help in the decision-making when the indication for TAVI is uncertain because of the risk of futility due to frailty or relevant comorbidities and in certain cases of low-flow/low-gradient AS (3, 4).

AS represents a complex, multifaceted set of syndromes that may present in a range of manners and is not isolated to calcific degeneration of the aortic valve alone. Each component part of the system, from the ventricle proximal to the valve to the vasculature distal, can impact signs and symptoms (5–8). With such a more inclusive perspective, the indication for and timing of TAVI could be enhanced, adding precision to the decision-making process.

A previous study by our group identified a series of variables related to the aortic valve, left ventricle, cardiac rhythm, and arterial pulse wave that showed a high predictive value for functional recovery in patients undergoing TAVI (9).

Continuous cardiac afterload monitoring based on a combined analysis of flow velocity signal recorded by Doppler and aortic pressure, the velocity–pressure (VP) loops, has been suggested. This analysis appeared to provide insights into arterial mechanics with standard hemodynamic signals recorded in the operating room (10–12).

We designed an original diagnostic approach based on the integrated analysis of left ventricular outflow tract flow velocity–aortic pressure. In this study, we aimed to assess the value of this approach to predict the objective functional improvement after TAVI and thus to estimate the AS burden.

## Methods

### Population

The present study was performed in a subgroup of population included in a larger study previously published by our group (9).

All consecutive patients scheduled for TAVI in our institution who met the inclusion criteria were prospectively included in the study. The criteria for inclusion are as follows: (1) diagnosis of symptomatic severe AS (according to guidelines) without significant regurgitation, (2) indication for TAVI established by the institutional Heart Team, and (3) undergoing a TAVI procedure through femoral artery access. Patients who did not consent or who exhibited cognitive impairment that prevented them from properly understanding the investigational procedures were excluded.

Patients who were initially included but presenting severe periprocedural complications such as coronary obstruction, annulus rupture, or stroke were finally excluded from the analysis, given their relevant effect on physiologic measurements and functional recovery after the procedure. For the same reason, patients who required permanent pacemaker implantation after the procedure and showed pacemaker dependence were excluded.

All the procedures were performed in the appropriate setting of a catheterization laboratory dedicated to structural heart interventions. The local TAVI program was started in 2009 and was mostly based on balloon-expandable prosthetic valves. The study was approved by the corresponding Institutional Review Board, and all participating patients signed the informed consent after proper explanation of the investigational procedures. Database was completely anonymized.

All patients were monitored in a specific structural cardiology office, where the clinical follow-up was performed, tests were applied, and the medical treatment of each patient was optimized as much as possible.

### Pre-procedural and post-procedural clinical and functional evaluation

The workflow of the study is shown in Supplementary Figure 1. Once the patients received the indication for TAVI by the Heart Team, they were evaluated in the outpatient office for structural heart interventions. In this visit, all the clinical information was collected, and the functional status of the patient including quality of life and frailty was assessed using accepted questionnaires [SF-36, EQ-5D, Barthel I, Essential Frailty Toolset, NYHA class, and the Kansas City Cardiomyopathy Questionnaire (KCCQ)], a 6-min walk test, and the determination of NT-proBNP (N-terminal pro-brain natriuretic peptide) levels in the baseline condition. All clinical and functional assessment was repeated 30 days, 6 months, and 12 months after the TAVI procedure by the same team and in the same setting. Transthoracic echocardiography was performed before TAVI and in subsequent visits after TAVI.

### Intraprocedural investigational examinations

A systematic protocol-specific transesophageal and transthoracic echocardiographic examination was performed during the TAVI procedure in all patients before and after prosthetic valve implantation. The intraprocedural echocardiographic examinations were performed simultaneously with the invasive central pressure measurements. The following parameters are obtained: left ventricular ejection fraction (LVEF), stroke volume, maximal and mean aortic valve gradients, energy loss index, pulse Doppler recording at the left ventricular outflow tract (LVOT), and continuous Doppler recording through the aortic valve. The Doppler recordings were generally obtained from the transthoracic echocardiography except for those few cases in which the transgastric view provided a more adequate recording.

Invasive pressure measurements in the ascending aorta were performed with a 5- or 6-Fr pigtail catheter attached to a fluid-filled manometer system. The catheter was inserted through a femoral 6-Fr introducer sheath (contralateral to the femoral access for TAVI) with its tip steadily positioned in the middle portion of the ascending aorta, at least 3 cm over the cusps of the aortic valve. Invasive aortic pressure recordings were taken at two different intraprocedural moments, pre- and post-TAVI, simultaneously with the echocardiographic examinations. An average of 20 cardiac cycles was used to render the final pressure measurements.

### Construction and analysis of the flow velocity in LVOT–pressure in ascending aorta loops

**Figure 1:**
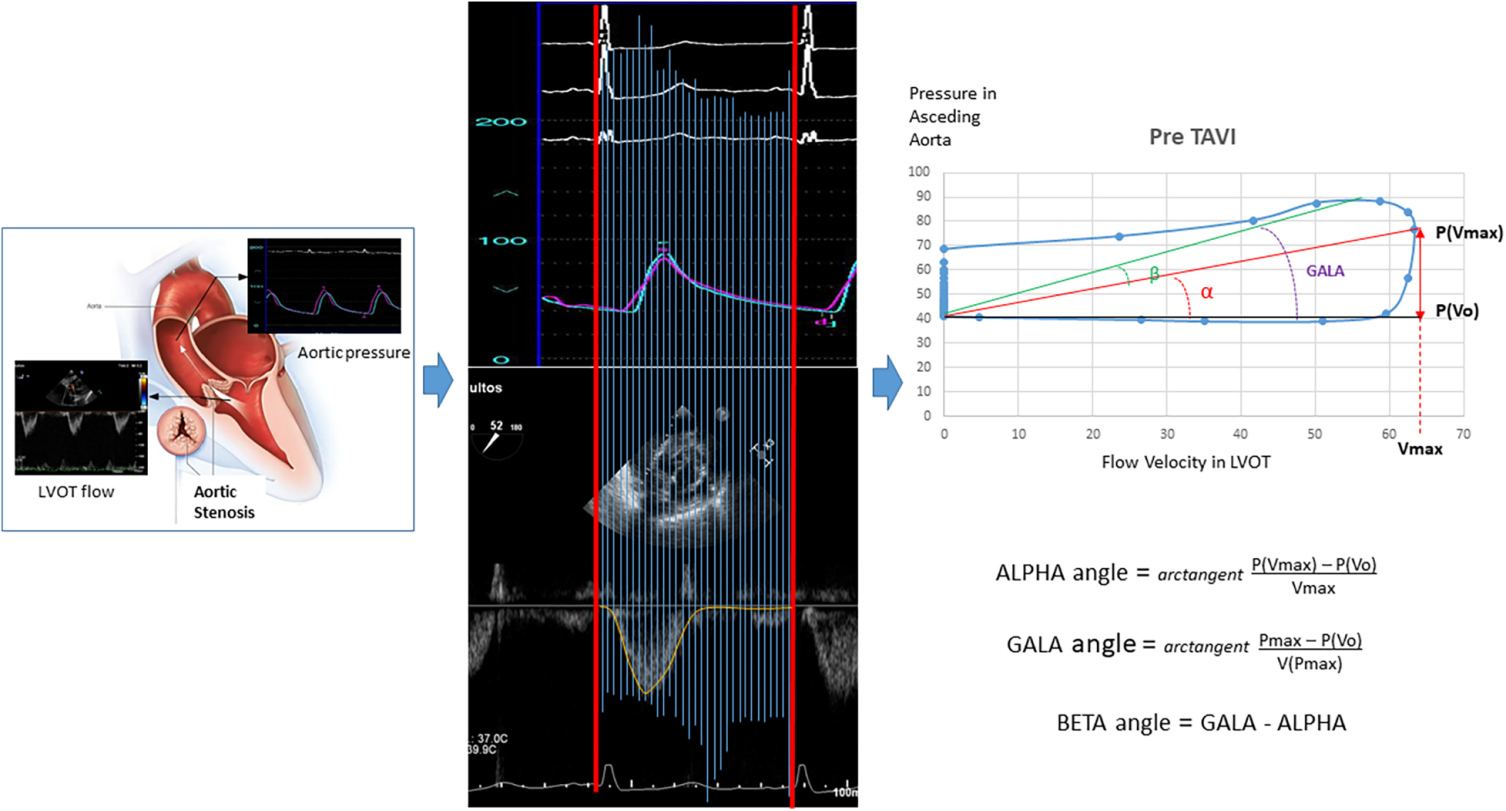
Construction and analysis of the loops for “flow velocity in left ventricular outflow tract/blood pressure in the aorta.” LVOT, left ventricular outflow tract.

The first step was to align both simultaneous VP records and create a mesh to obtain the same number of coincident points in time for velocity and pressure (Figure 1). The resulting images were processed using the WebPlotDigitizer application (13). The resulting data were saved in two independent vectors to later face them and create the loops with a program developed in R. From the loops, the angles and distances between the points defined by the maximum and minimum pressure–velocity values were calculated.

### Endpoints and definitions

The objective functional improvement of a patient with AS after the TAVI procedure was defined as the achievement at 6 months of an increase of at least 10% in the distance covered during the 6-min walk test or a reduction of at least 50% in NT-proBNP blood levels with respect to pre-TAVI when this 10% increase was not evident. In this way, with both criteria, the potential presence of factors that limit the speed of gait and are not related to cardiovascular capacity was taken into consideration.

For those patients who died before the 6-month landmark and after the 30-day evaluation, improvement was based on this evaluation, but this was considered negative if the patients suffered from or died of heart failure afterward. Patients who died before the 30-day follow-up, patients who died because of heart failure were considered without improvement, and the rest of the patients who died from other causes were excluded from the analysis since no functional evaluation was available. Subjective improvement was considered if patients reported a positive change in at least one class of the NYHA classification and/or an increase in at least 10 points in the KCCQ. All patients underwent a systematic clinical follow-up at 2 years. Baseline staging of cardiac damage was conducted according to the classification proposed by Généreux et al. (14).

### Statistical analysis

Continuous variables are presented as means ± standard deviations or medians (interquartile ranges) according to the type of distribution, and categorical variables are presented as percentages. Distribution was assessed for each variable with the Shapiro–Wilk test. Accordingly, continuous variables were compared using the Student's *t*-test if they followed a normal distribution and by non-parametric tests when this was not the case. The categorical variables were compared with the chi-squared test or Fisher’s exact test, as required.

Multivariable logistic regression analysis identified independent predictors of objective functional improvement post-TAVI. Among all the clinical, echocardiographic, hemodynamic, and VP loop-related variables, those that showed a univariate relationship with an outcome (*p *< 0.2) were entered into the multivariable logistic regression model. Then, a stepwise elimination analysis was performed to define a useful subset of predictors.

The Hosmer–Lemeshow test or the likelihood ratio test was used to evaluate the goodness of fit, that is, the overall significance of the model. The Nagelkerke *R*^2^ was used to determine the amount of variance of the dependent variable, which explains the estimated model. This indicates the degree of usefulness of the independent variables in predicting the dependent variable. When using prediction models, it is first necessary to differentiate two subsets of the original sample. One will be used to estimate the desired model (train data), and the other will be used to test the estimated model (test data). The R createDataPartition command allowed us to obtain these sets from the original sample.

In the presence of unbalanced samples, it is necessary to balance the set with which the model is to be estimated, that is, the train data set. To solve this problem, R function ROSE (*Random Over-Sampling Examples*), which allowed us to deal with binary classification problems in the presence of unbalanced samples, was used. Because the sample partitions used for the model estimation phase were random and different from each other, they led to different estimates of the prediction model. That is why, to finish with the model estimation phase, it is necessary to carry out machine learning techniques known as assembled models. There are different ways to obtain the assembled model, either by using different algorithms or by varying the training data obtained by bootstrapping, for the same algorithm. In this last procedure, each model has different parameters since it has been estimated from a different sample in each case. This is the method that has been chosen to solve the problem that was raised above. Assembled models are used to more robustly predict an outcome from multiple models.

The discriminating power of the parameters for predicting objective functional improvement was assessed by considering the area under the curve from the receiver operating characteristic (ROC) analysis. Box and whisker plots were built to show the baseline and post-TAVI evolution of variables according to improvement subgroups. Kaplan–Meier curves for event-free survival were obtained for each group and compared using the log-rank test and the hazard ratios with 95% confidence intervals. *p-*values of <0.05 were considered statistically significant. Statistical packages SPSS 25.0, R programs, and MedCalc Statistical Software version 19.6.4 (MedCalc Software Ltd., Ostend, Belgium) were used during the course of the study.

## Results

Finally, among the 105 eligible patients, 102 consecutive patients who underwent TAVI and met the inclusion criteria were included in the study. The baseline characteristics of the patients are listed in Supplementary Table S1. Approximately 52% were women, and their mean age was 81 ± 6.6 years. Among these patients, 82 (80.4%) presented objective functional improvement 6 months after the intervention, whereas 93 (91%) reported a variable degree of subjective improvement. The changes in 6-min walk test results and NT-proBNP levels observed after the procedure are shown in Supplementary Figure S2. The group with objective improvement significantly increased the distance in the walk test and showed a significant decrease in biomarker levels. The group without improvement experienced no positive changes in these parameters.

The hemodynamic, echocardiographic, and VP loop-derived parameters at baseline for both groups, with and without objective functional improvement, are listed in Table 1. A lower central systolic blood pressure (SBP) was significantly associated with clinical improvement at 6 months. The variables that reflect characteristics of the arterial system, such as pulse pressure (PP), valve–aortic impedance (Zva), or total arterial distensibility (SVi/PP), did not show significant differences between the groups.

**Table 1:** Baseline parameters according to objective functional improvement after TAVI.

	No improvement	Improvement	*p*
	*n* = 20	*n* = 82	
SBP (mmHg)	136 ± 4	130 ± 3	0.03
MAP (mmHg)	95 ± 3	92 ± 2	0.16
PP (mmHg)	59 ± 4	58 ± 2	0.34
SVi/PP (ml/mmHg)	0.75 ± 0.23	0.8 ± 0.3	0.60
LVEF (%)	57 ± 2	56 ± 1	0.20
SVi (ml/kg/m^2^)	42 ± 3	43 ± 2	0.46
Aortic maximal gradient (mmHg)	81 ± 6	86 ± 3	0.51
Aortic mean gradient (mmHg)	48 ± 4	49 ± 2	0.67
Energy loss index (cm^2^/m^2^)	0.48 (0.4–0.63)	0.44 (0.35–0.5)	0.29
Indexed aortic valve area (cm^2^/m^2^)	0.42 (0.34–0.54)	0.4 (0.32–0.5)	0.26
Zva (mmHg/ml/m^2^)	4.6 ± 1.3	4.7 ± 1.7	0.87
VP analysis			
ALPHA angle (°)	4.21 (0.6–8)	9.6 (3.9–17)	0.027
BETA angle (°)	77.3 (23.63–86.8)	54.7 (25.4–84)	0.26
GALA angle (°)	80.78 (36.5–90)	64.6 (48.1–90)	0.61
P(Vmax) − P(Vo)	8.59 ± 1.9	16.6 ± 1.8	0.004
Vmax	83.01 ± 5.6	80.88 ± 3.3	0.78
Pmax − P(Vmax)	47 ± 27	35 ± 18	0.09
P(Vmax) − P(Vo)/Vmax	0.09 ± 0.08	0.23 ± 0.19	0.001

MAP, mean arterial pressure; Zva, valvuloarterial impedance, defined as (SBP + mean aortic gradient)/SVi.

Values are mean ± SD or medians (25th–75th interquartile ranges), depending on variable distribution.

The baseline values of the parameters derived from the VP loops are also listed in Table 1. A larger ALPHA angle or the closely related parameters “(Pressure at Vmax − Pressure at Vo)/Vmax” and “P(Vmax) − P(Vo)” were all significantly associated with objective functional improvement at 6 months.

**Figure 2:**
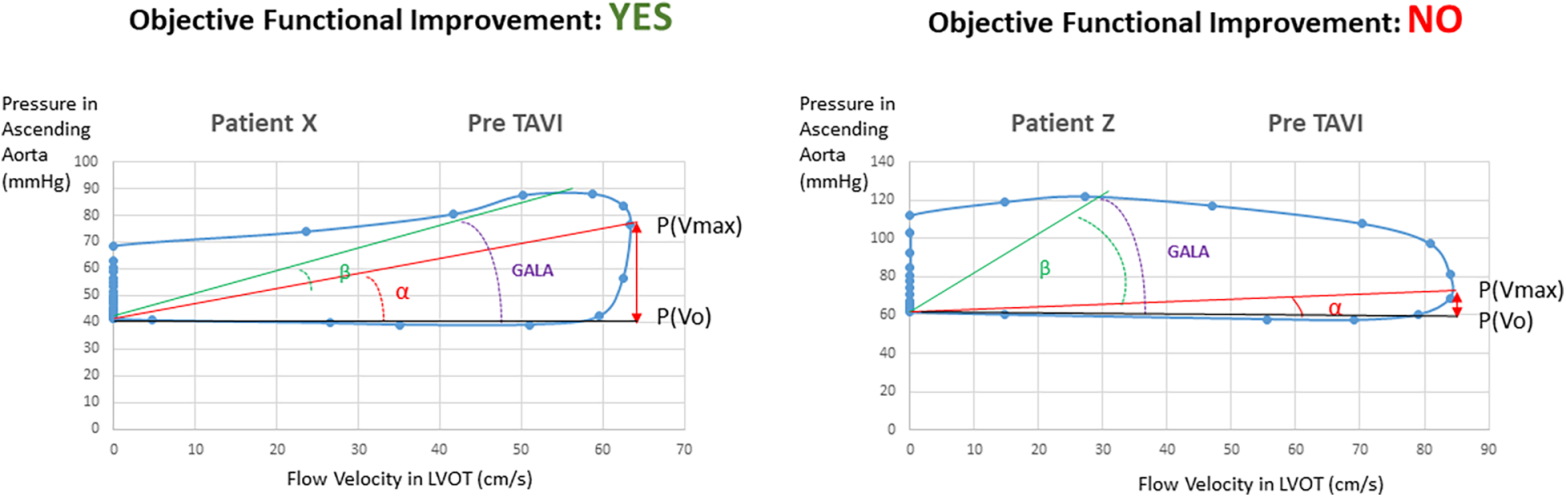
Velocity–pressure loops at baseline in patients with and without objective functional improvement after TAVI.

The respective VP loops of patients with and without objective functional improvement, showing the remarkably larger values for the ALPHA angle and the P(Vmax) − P(Vo) difference in the patients experiencing improvement, are illustrated in Figure 2.

In the multivariate regression analysis, the only variable that resulted in an independent predictor of objective functional improvement was the ALPHA angle (OR 1.12, 95% CI 1.0064–1.2417; *p* = 0.03). Replacing the ALPHA angle value for the equivalent parameter, “P(Vmax) − P(Vo)/Vmax,” resulted in this being a unique independent predictor.

**Figure 3:**
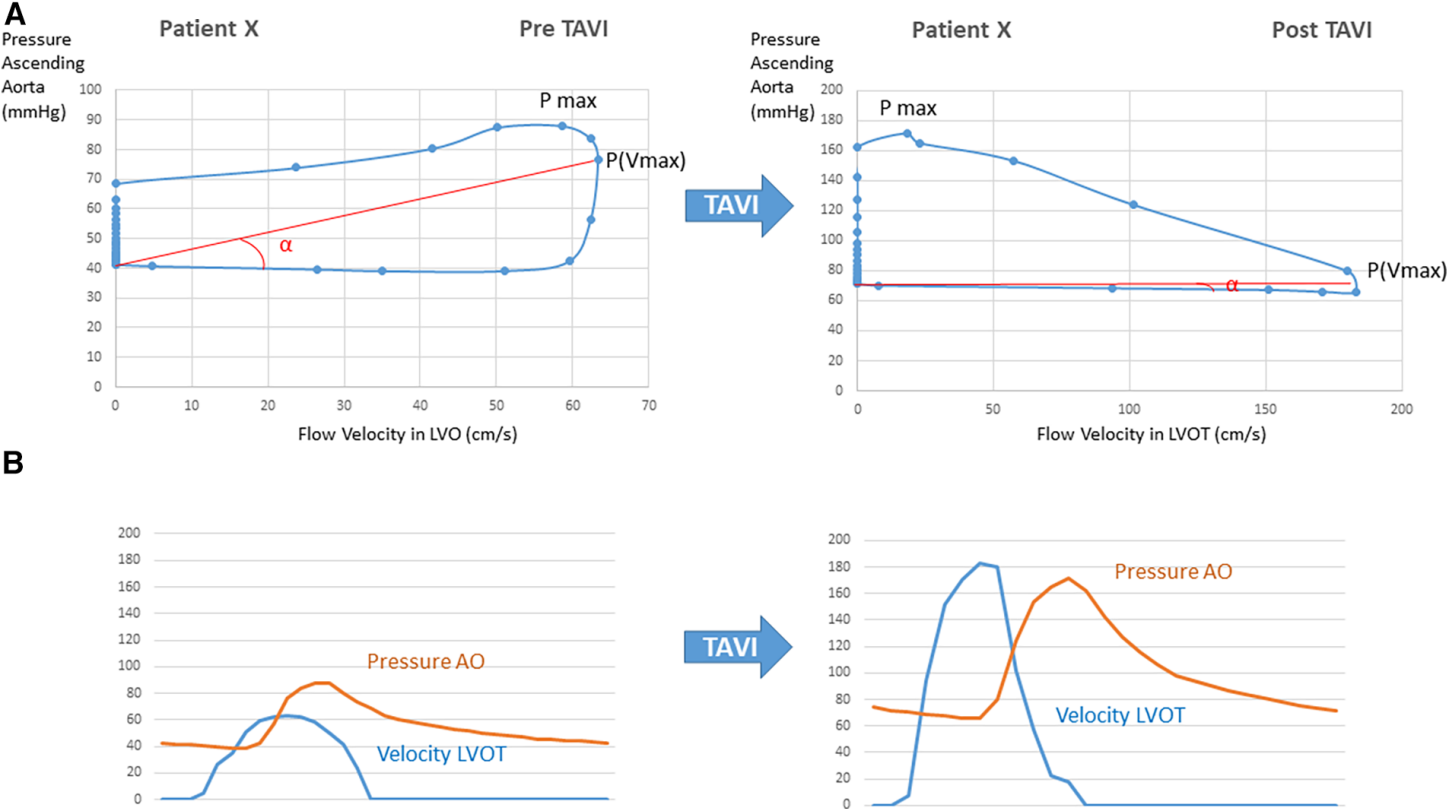
(**A**) Velocity–pressure loops at pre- and post-TAVI stages in a patient with significant objective functional improvement after the procedure. (**B**) Corresponding recordings for pressure and velocity showing changes in magnitude and time coupling after TAVI.

The proportional changes observed for the different variables analyzed in the study after the TAVI procedure according to the reported objective improvement are presented in Table 2. The VP loops of one patient at baseline and after TAVI, in whom objective functional improvement was noted, are illustrated in Figure 3. The corresponding recordings for pressure and velocity showed changes in magnitude and time coupling after TAVI.

**Table 2:** Proportional variation of parameters from baseline to post-TAVI according to objective functional improvement.

	No improvement	Improvement	*p*
	*n* = 20	*n* = 82	
Variation pre–post			
SBP	0.29 ± 0.26	0.37 ± 0.27	0.32
MAP	−0.08 ± 0.14	−0.03 ± 0.16	0.25
PP	−0.14 (−0.26/0.01)	−0.06 (−0.24/0.1)	0.55
Aortic maximal gradient	−0.7 (−0.77/−0.61)	−0.73 (−0.8/−0.62)	0.36
Aortic mean gradient	−0.76 (−0.8/−0.69)	−0.78 (−0.83/−0.70)	0.49
SVi	0.08 (−0.02/0.24)	0.05 (−0.13/0.39)	0.55
Zva	−0.30 (−0.46/−0.16)	−0.28 (−0.46/−0.12)	0.55
ALPHA angle	0.09 (−0.45/0.71)	−0.60 (−0.84/0.28)	0.03
BETA angle	−0.26 (−0.40/0.86)	−0.01 (−0.29/1.04)	0.37
GALA angle	−0.19 (−0.35/0.52)	−0.05 (−0.35/0.56)	0.76
Pmax − P(Vmax)	0.34 ± 0.9	1.43 ± 2.7	0.006
P(Vmax) − P(Vo)	0.23 (−0.38/1.55)	−0.50 (−0.78/0.94)	0.04

The proportion of variation was calculated as (value post − value pre)/value pre. Values are means ± SDs or medians (25th–75th interquartile ranges), depending on the variable distribution.

**Figure 4:**
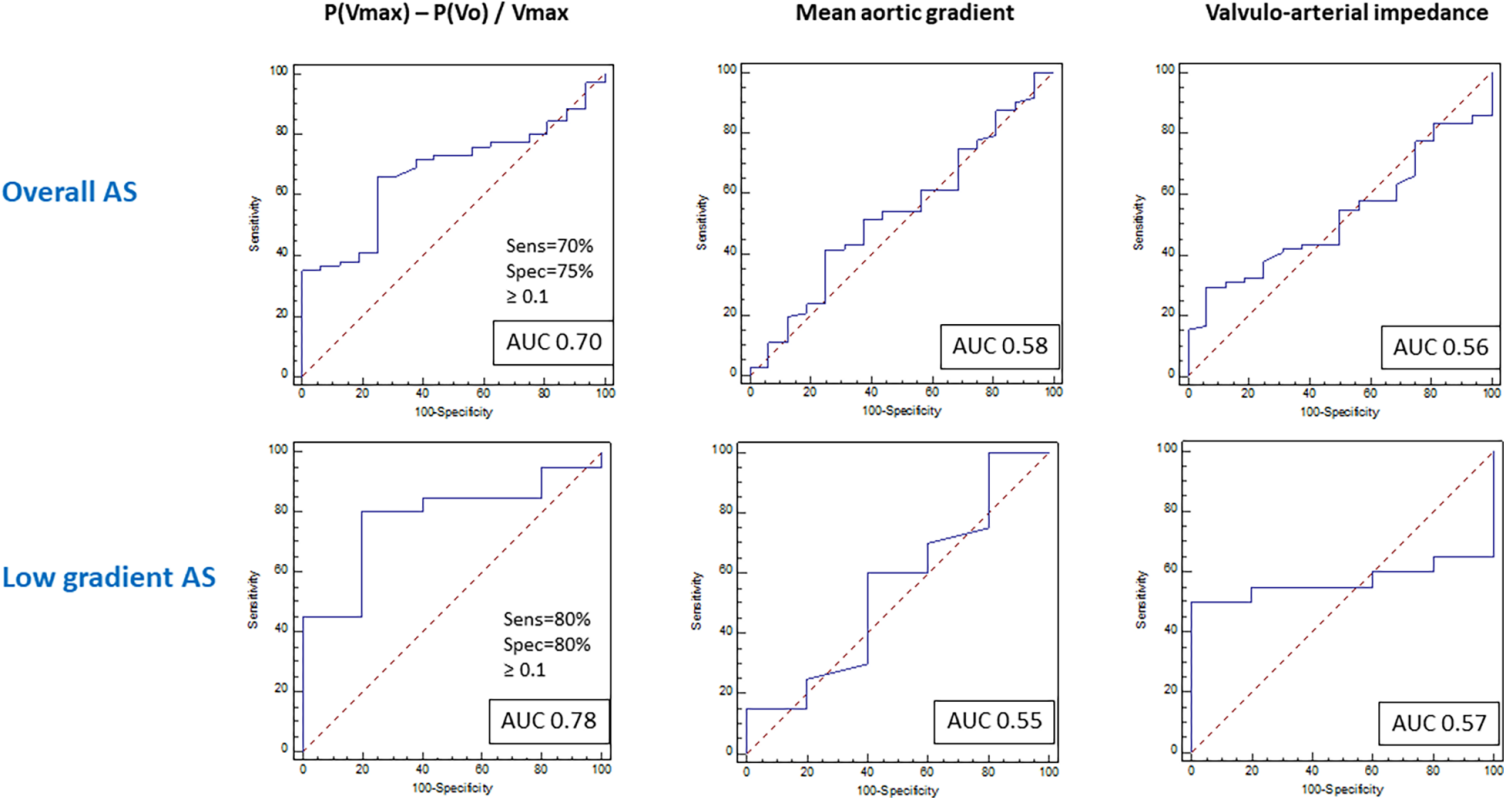
Receiver operating curves for prediction of functional improvement after TAVI in the general population with aortic stenosis and the low-gradient aortic stenosis subpopulation.

The discriminative performance of different parameters to predict the objective functional improvement after TAVI is described in Figure 4 and Supplementary Table S2. The AUC was significantly higher for the ALPHA angle and [P(Vmax) − P(Vo)]/Vmax and also for the closely related P(Vmax) − P(Vo) compared with the other conventional parameters used in clinical practice to estimate the severity of AS. These differences were even more pronounced in the low-gradient AS subgroup, most of them with a low-flow condition (≤35 ml/m^2^). The cutoff value for [P(Vmax) − P(Vo)]/Vmax was 0.1 in the overall and low-gradient groups.

**Figure 5:**
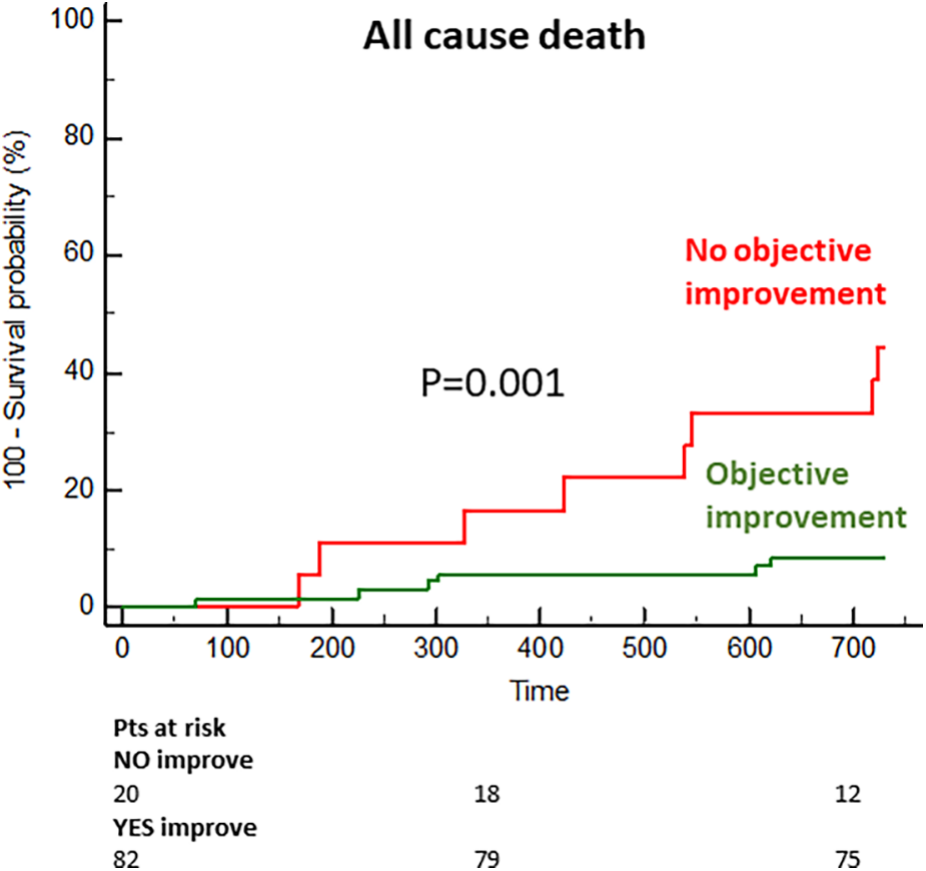
Cumulative incidence of mortality in the subgroups with and without objective functional improvement.

In the long term, the group with objective functional improvement showed a significantly lower incidence or mortality (9% vs. 44% at the 2-year follow-up; *p* = 0.001) (Figure 5).

The predictive value of the VP loop-derived parameter [P(Vmax) − P(Vo)]/Vmax was assessed in a validation cohort of 119 patients. The clinical and procedural characteristics of the validation cohort are described in Supplementary Tables S3 and 4. Applying the same definition for objective functional improvement after TAVI, the parameter showed an AUC of 0.67 for the total cohort and 0.76 for the low-gradient subgroup (both *p* < 0.001).

**Figure 6:**
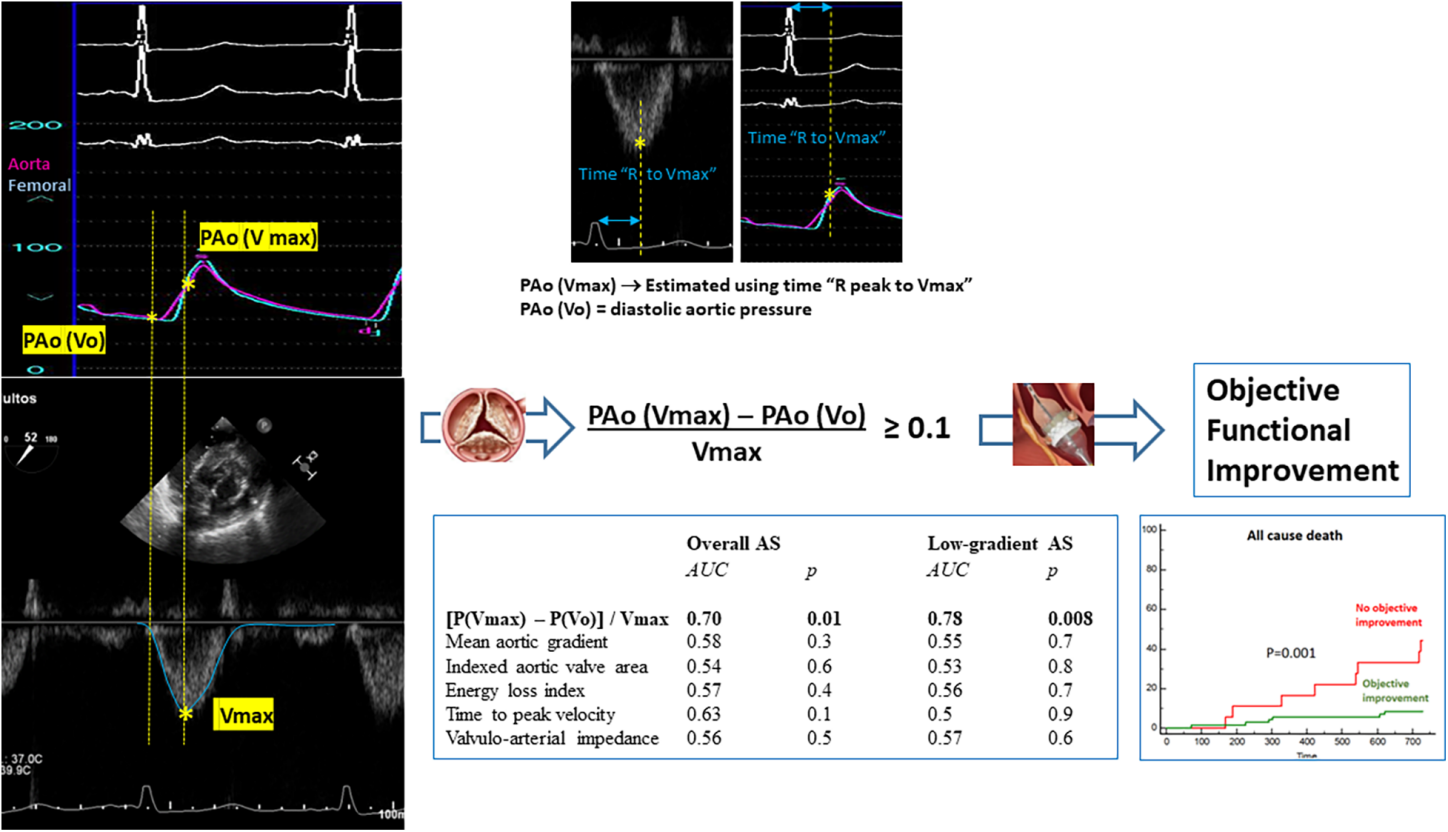
Parameter “(Pressure at Vmax − Pressure at Vo)/Vmax” derived from the integrated analysis of flow velocity in the left ventricular outflow tract and blood pressure in the aorta at baseline results in a more accurate predictive factor for objective functional improvement after TAVI in the general population with aortic stenosis and the low-gradient aortic stenosis subpopulation. The calculation of this parameter is simplified through the electrocardiographic recordings in simultaneous pressure and Doppler tracings. The objective functional improvement after TAVI portends a highly relevant prognostic effect.

The summarized results are graphically illustrated in Figure 6.

## Discussion

In the present study, the parameters derived from the integrated analysis of LVOT flow velocity and ascending aorta pressure resulted in the only independent predictors for objective clinical improvement at 6 months in patients with severe aortic stenosis undergoing TAVI.

Namely, the parameter “[P(Vmax) − P(Vo)]/Vmax” resulted in a predictor for objective improvement in the overall population and was even more accurate in the low-gradient subgroup. This parameter outperformed all other conventional parameters used in clinical practice to estimate AS severity, particularly in the challenging subgroup of patients with low-gradient AS.

The calculation of this parameter is not complex since both aortic pressure and flow velocity recordings include a simultaneous electrocardiographic recording in the same lead. Thus, the pressure at Vmax is estimated through the time between peak R-wave and peak Doppler flow velocity applied to the pressure curve, and the pressure at Vo is equivalent to the diastolic pressure. We do not have a clear pathophysiological explanation for the relationship between this parameter and the true hemodynamic load imposed by aortic stenosis (estimated from the functional improvement patients experience after valve replacement). However, we are convinced that this parameter reflects the interaction, throughout the ventricular ejection phase, between the flow velocity proximal to the valve (at the LVOT) and the dynamics of pressure just distal to the valve (aortic root). This multidimensional integral nature, which contemplates pressure, velocity, and time, explains its additional value with respect to pressure parameters such as the gradient, which are unidimensional.

Vallée and colleagues described aortic velocity–pressure loops and a series of angles derived from them (ALPHA, BETA, and GALA) as an estimate of ventriculoaortic coupling that is easily monitored during surgical interventions, obtaining a continuous measurement of left ventricular afterload (10–12). In the study by Hong et al., to build the VP loop in the ascending aorta, the aortic velocity was measured immediately after the acquisition of invasive pressures at the center of the LVOT with close attention paid to obtaining an angle of the Doppler signal to aortic blood flow close to 0° (11). Noteworthy, in studies aimed to measure the valvuloarterial impedance in patients with AS using magnetic resonance imaging, flow measurement is routinely performed at the LVOT or just above the valve (15, 16). The flow measurement in the LVOT, where complex flow is less prominent, is thought to provide a more accurate measurement of forward flow (17).

In our study, an original new design was proposed to assess the effect of aortic stenosis in which flow is measured in the LVOT and pressure in the ascending aorta, so it is plausible that the ALPHA angle or [P(Vmax) − P(Vo)]/Vmax could be largely representing the effect of the valvular component of the afterload. Thus, patients with clinical improvement at 6 months had significantly higher values at baseline than those who did not improve.

The conventional echocardiographic parameters such as the indexed aortic valve area, energy loss index, and peak or mean aortic gradients were not significant predictors. The delayed time to peak velocity also did not show any independent predictive value (18).

Briand et al. described the concept of valvuloarterial impedance (Zva) to quantify the global afterload in aortic stenosis since this variable took into account the valve and arterial load components (5). However, the valvuloarterial impedance did not result in an independent predictor when entering the VP loop parameters in the regression model. Furthermore, its predictive value was poor compared with the VP loop-derived values.

Patients with low-gradient/low-flow AS, particularly with low ejection fraction, have a significantly worse medium-term to long-term survival compared with all other patients undergoing TAVI (19). In this setting, a low-dose dobutamine stress echocardiography is recommended to distinguish between true severe and pseudosevere aortic stenosis. However, after TAVI, the absence of contractile reserve at baseline in this test was not associated with any negative effect on clinical outcomes or LVEF changes at follow-up (19). Cardiac tomography assessment of the degree of valve calcification provides important additional information (20). Nonetheless, given the poor prognosis with medical treatment, TAVI should be considered an option in certain patients with low-gradient AS. Therefore, it is important to know the parameters that allow identifying patients who may benefit from the intervention.

In our study, the patients with low-gradient AS who underwent TAVI had had diagnostic confirmation of the severity of the stenosis, in some cases after dobutamine stress echo or after considering a high degree of valve calcification on tomography. Nonetheless, in these patients, valve calcification had no predictive value for functional improvement after TAVI. Remarkably, the VP loop parameters showed a notable discriminatory value in the low-gradient population regardless of the stroke volume, clearly better than the yielded by valve-related echocardiographic measurements or the valvuloarterial impedance.

The contribution of this study is original and provides an additional value with respect to the previous study of our group since this study investigates the value of an integrated analysis of LVOT flow velocity and aortic pressure, measured non-invasively and invasively, respectively, in the assessment of aortic stenosis. This true novel approach could have by itself a high potential value in defining the hemodynamic burden imposed by aortic stenosis. In contrast, the previous study included a series of conventional parameters well known in clinical practice (9). Thus, the new proposed method seems to outperform any of those conventional parameters.

These findings should be prospectively validated in a larger population of patients currently treated with TAVI. The development of software capable of facilitating the integrated analysis of pressure and Doppler tracings would be welcome. In addition, the use of central pressure recordings estimated by non-invasive techniques would allow a broader implementation of this type of analysis (8, 21).

## Limitations

This study contains several limitations. First, the sample size of the cohorts limits the statistical power of the study. The fact that most patients presented clinical improvement at 6 months has meant that the sample is unbalanced, which required machine learning techniques to try to correct the regression analysis outcomes. Anyway, the predictive superiority of the VP loop parameters over the conventional metrics used to estimate AS severity was evident in this study. In addition, the predictive power of these parameters was prospectively validated.

The definition of objective functional improvement was specific to the study and, although well thought out, may be questionable. However, it was sufficiently precise and, at the same time, conservative, as confirmed by seeing how the group considered without improvement showed even worse post-TAVI performance in the walking test and the absence of changes in heart failure biomarkers. In addition, the classification also showed important prognostic implications. The assessment time of 6 months can be discussed; however, we know from previous studies that the improvement after TAVI is rapid, being evident even at 30 days (22, 23). On the other hand, a later evaluation, especially in an elderly population, may be affected by the concurrence or progression of other unrelated pathological processes, such as coronary artery disease or certain comorbidities.

These results are certainly applicable to the profile of patients included in the study, who are patients with degenerative-calcified aortic stenosis, most of whom have tricuspid anatomy and a minority have bicuspid anatomy. Therefore, the results could obviously be applicable to patients undergoing aortic valve replacement surgery. The validity of the method in bicuspid stenosis or in rheumatic stenosis could be defended taking into account that the pathophysiological effects of aortic valve stenosis would be comparable.

In methods, it is indicated that patients who were initially included but presenting severe periprocedural complications such as coronary obstruction, annulus rupture, or stroke were finally excluded from the analysis, given their relevant effect on physiologic measurements and functional recovery after the procedure. The aim of the study was to establish pre-procedural predictors of objective functional improvement since these are the ones that would be of value to help in the decision-making when the indication for TAVI is uncertain. With regard to comorbidity and frailty, all were included in the predictive model. The fact is that the clinical selection process prior to indication already excludes those with very high frailty or severe comorbidities that seriously compromise the patient's short-term future and make the transcatheter aortic valve implantation (TAVI) procedure futile.

The rate of missing data was extremely low. This is a series of limited sizes, from a single center with a systematic prospective database. All patients belong to our regional public health system and are therefore perfectly traceable. In addition, all patients in the TAVI program are followed up in our department.

The application of this method in clinical practice would require the use of specific software, but it is not complex and could be developed without great difficulty.

## Conclusion

The integrated analysis of the left ventricular outflow tract flow velocity and aortic pressure allows us to predict the degree of objective functional improvement after TAVI and thus to estimate the aortic stenosis burden more precisely than the conventional parameters used to assess the severity of aortic stenosis, particularly in low-gradient patients.

## Data Availability

The raw data supporting the conclusions of this article will be made available by the authors, without undue reservation.

## Supplementary material

Supplementary Figure S1 Protocol for the workflow of the study. TAVI = transcatheter aortic valve implantation; TT = transthoracic; TE = transesophageal.

Supplementary Figure S2 Baseline and 6 months post-TAVI values for the 6-minute walk test and for blood levels of NT-proBNP (Nitro-terminal-pro brain natriuretic peptide), according to the classification for objective functional improvement.
