# Supplementary material for: First description and validation of a new method for estimating aortic stenosis burden and predicting the functional response to TAVI
Source: Front Cardiovasc Med. 2023 Nov 14;10:1215826. doi: 10.3389/fcvm.2023.1215826 (PMC10682652; doi:10.3389/fcvm.2023.1215826)
Supplement: Supplementary file 1 [file Table1.docx]

**Supple Table 1. Baseline characteristics and procedural data**

**N = 102**

Age, years 81 ± 6.6

Female gender 53 (52%)

Diabetes 37 (36.2%)

High blood pressure 84 (82.3%)

Dyslipidemia 67 (66%)

Coronary artery disease 33 (32.3%)

Previous MI 7 (6.8%)

Previous PCI 22 (21.6%)

< 6 months 10 (9.8%)

Previous CABG 1 (0.9%)

Carotid disease 2 (1.9%)

Peripheral vascular disease 6 (5.8%)

Atrial fibrillation 41 (40%)

Previous pacemaker 8 (7.8%)

Chronic renal failure

GFR < 60 ml/min 46 (45%)

GFR < 30 ml/min 5 (4.9%)

EuroSCORE II 3.1±2

STS-score mortality 3.5±1.9

Agatston calcium score 2884 (1765-3923)

**Echocardiographic data**

Left ventricular ejection fraction (%) 60 (55-63)

Stroke volumen index (ml/m^2^) 44.4±17.6

Interventricular septum (mm) 14.7±3.5

Posterior wall (mm) 15±2.7

Maximal aortic gradient (mmHg) 83 (66-100)

Mean aortic gradient (mmHg) 49 (39-59)

Aortic valve área (cm^2^) 0.69 (0.54-0.85)

Indexed aortic valve área (cm^2^/m^2^) 0.38 (0.3-0.5)

Energy loss index (cm^2^/m^2^) 0.77 (0.54-0.93)

LVOT velocity/Aortic valve velocity 0.2 (0.15-0.25)

Valvuloarterial impedance (Zva) (mmHg/ml/m^2^) 4.5 (3.5-5.4)

Delayed time to peak velocity, ms 114±16

**Symptomatic and functional status**

NYHA

Class I 0

Class II 62 (60.8%)

Class III 38 (37.2%)

Class IV 2 (1.9%)

KCCQ 65 ±13.4

Test SF-36 46 (34-63)

Test EQ-5D 50 (50-70)

Barthel index 100 (95-100)

Charlson comorbility index 5 (4-6)

Essential Frailty Toolset

0 34 (33.3%)

1-2 57 (55.8%)

3-4 11 (10.7%)

5 0

Staging cardiac damage

0-1 14 (13.7%

2 46 (45%)

3-4 42 (41%)

**Procedure**

Balloon expandable valve prosthesis 102 (100%)

Size of valve prothesis

23mm 37 (36.2%)

26mm 53 (52%)

29mm 12 (11.7%)

Femoral TAVI Access site 102 (100%)

Predilatation 60 (58.8%)

Postdilatation 7 (6.8%)

Values are n (%), mean ± SD, or median (25th-75th interquartile range), depending on variable distribution.

MI: Myocardial infarction; PCI: Percutaneous coronary intervention; CABG: Coronary artery bypass graft; GFR: Glomerular filtration rate; STS-score mortality: Society of Thoracic Surgeons score of mortality; NYHA: New York Heart Asocciation; KCCQ: Kansas City Cardiomiopathy Questionnarie; SF-36: The Short Form-36 Health Survey; EQ-5D: European Quality of life 5 Dimensions; LVOT: Left ventricular outflow tract;; Zva: Valvuloarterial impedance, defined as: (SBP + mean aortic gradient) / stroke volume index.

**Suppl Table 2. Performance of different parameters for prediction of objective functional improvement after TAVI.**

**Overall AS Low-gradient AS**

**AUC p best cutoff AUC p best cutoff**

ALPHA angle 0.70 0.005 5 0.78 0.007 5

[P(Vmax) – P(Vo)] / Vmax 0.70 0.01 0.1 0.78 0.008 0.1

P(Vmax) – P(Vo) 0.68 0.01 7 0.73 0.03 6

Mean aortic gradient 0.58 0.3 0.55 0.7

Indexed aortic valve area 0.54 0.6 0.53 0.8

Energy loss index 0.57 0.4 0.56 0.7

Time to peak velocity 0.63 0.1 0.5 0.9

Valvulo-arterial impedance 0.56 0.5 0.57 0.6

AUC: area under the receiver operating characteristic curve.

**Suppl Table 3. Baseline clinical characteristics in validation cohort**

**N = 119**

Age,years 80 (76-85)

Female gender 62 (52%)

Diabetes 34 (28.6%)

High blood pressure 101 (84.8%)

Dyslipidemia 85 (71.4%)

Coronary artery disease 39 (32.7%)

Previous MI 11 (9.2%)

Previous PCI 25 (21%)

< 6 months 10 (8.4%)

Previous CABG 2 (1.7%)

Carotid disease 4 (3.4%)

Peripheral vascular disease 10 (8.4%)

Mitral valve disease 17 (14.2%)

Atrial fibrillation 41 (34.4%)

Previous pacemaker 4 (3.4%)

Chronic renal failure

GFR < 60 ml/min 54 (45.3%)

GFR < 30 ml/min 5 (4.2%)

EuroSCORE II 3.2 ± 2.4

STS-score mortality 3.4 ± 2

Agatston calcium score 3004 ± 1356

**Symptomatic and functional status**

NYHA

Class I 0

Class II 81 (68%)

Class III 33 (27.7%)

Class IV 5 (4.2%)

KCCQ 56 (51-70)

Test SF-36 44.2 ± 17

Test EQ-5D 53.9 ± 14.4

Barthel index 92 ± 12.3

Charlson comorbility index 5.3 ± 2

Essential Frailty Toolset

0 43 (36.1%)

1-2 63 (52.9%)

3-4 13 (11%)

5 0

Values are n (%), mean ± SD, or median (25th-75th interquartile range), depending on variable distribution.

MI: Myocardial infarction; PCI: Percutaneous coronary intervention; CABG: Coronary artery bypass graft; GFR: Glomerular filtration rate; STS-score mortality: Society of Thoracic Surgeons score of mortality; NYHA: New York Heart Asocciation; KCCQ: Kansas City Cardiomiopathy Questionnarie; SF-36: The Short Form-36 Health Survey; EQ-5D: European Quality of life 5 Dimensions

**Suppl Table 4. Procedural characteristics in validation cohort**

**N = 119**

Balloon expandable valve prosthesis 119 (100%)

Size of valve prothesis

23 mm 45 (37.8%)

26 mm 60 (50.4%)

29 mm 14 (11.8%)

Femoral access site 119 (100%)

Predilatation 50 (42%)

Postdilatation 6 (5%)

Coronary obstruction 1 (0.8%)

Valve embolization 0

Second valve implantation 0

Aortic annulus rupture 1 (0.8%)

Stroke 1 (0.8%)

Major vascular complications 3 (2.5%)
