# Supplementary figures and images for: First description and validation of a new method for estimating aortic stenosis burden and predicting the functional response to TAVI
Source: Front Cardiovasc Med. 2023 Nov 14;10:1215826. doi: 10.3389/fcvm.2023.1215826 (PMC10682652; doi:10.3389/fcvm.2023.1215826)

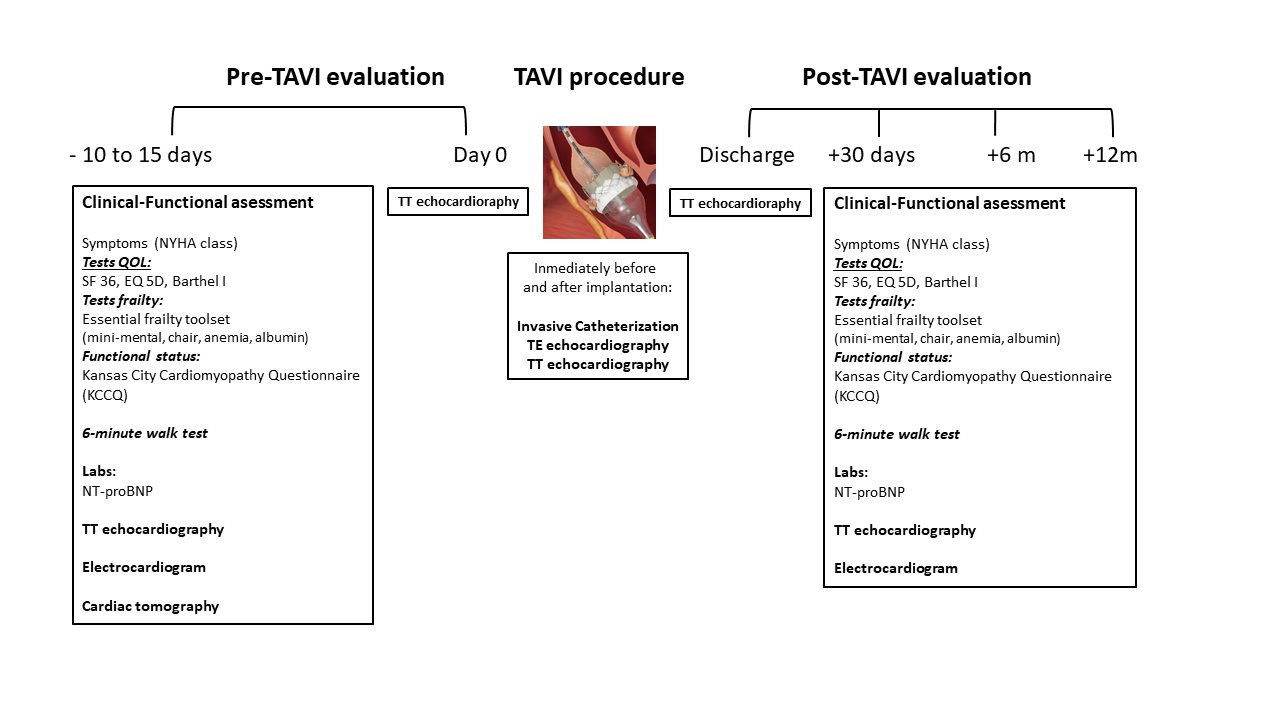

Supplement: Supplementary Figure S1 — Protocol for the workflow of the study. TAVI = transcatheter aortic valve implantation; TT = transthoracic; TE = transesophageal. [file Image1.tif]

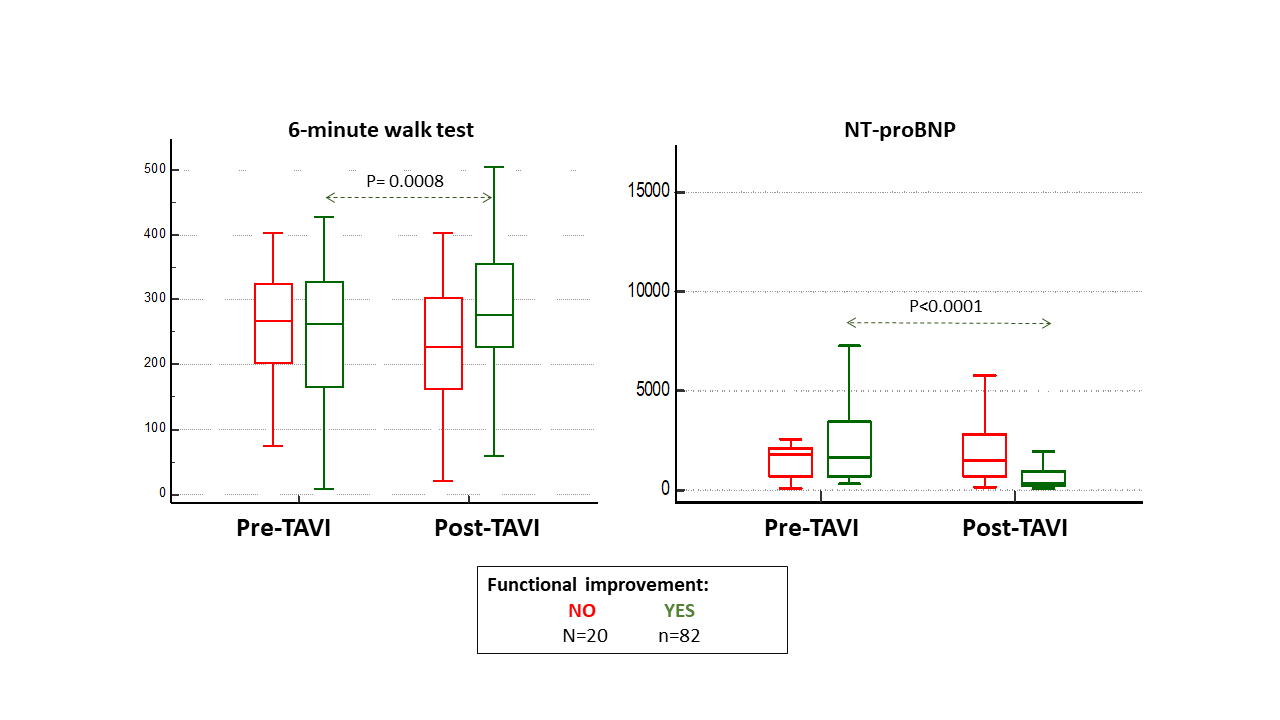

Supplement: Supplementary Figure S2 — Baseline and 6 months post-TAVI values for the 6-minute walk test and for blood levels of NT-proBNP (Nitro-terminal-pro brain natriuretic peptide), according to the classification for objective functional improvement. [file Image2.tif]
